# Quantifying acute kidney injury in an Ischaemia-Reperfusion Injury mouse model using deep-learning-based semantic segmentation in histology

**DOI:** 10.1242/bio.059988

**Published:** 2023-09-21

**Authors:** Andreea Luchian, Katherine Trivino Cepeda, Rachel Harwood, Patricia Murray, Bettina Wilm, Simon Kenny, Paola Pregel, Lorenzo Ressel

**Affiliations:** ^1^ Department of Veterinary Anatomy Physiology and Pathology, Institute of Infection, Veterinary and Ecological Sciences, Faculty of Health & Life Sciences, University of Liverpool, Liverpool, CH64 7TE, UK; ^2^ Department of Molecular Physiology and Cell Signalling, Institute of Systems, Molecular and Integrative Biology, University of Liverpool, Liverpool, L69 7BE, UK; ^3^ Centre for Pre-clinical Imaging, Institute of Systems, Molecular and Integrative Biology, University of Liverpool, Liverpool, L69 7TX, UK; ^4^ Department of Paediatric Surgery, Alder Hey in the Park, Liverpool, L14 5AB, UK; ^5^ Department of Veterinary Sciences, University of Turin, Turin, 8-10124, Italy

**Keywords:** Deep-learning, Ischaemia-reperfusion injury, Mouse, Kidney

## Abstract

This study focuses on ischaemia-reperfusion injury (IRI) in kidneys, a cause of acute kidney injury (AKI) and end-stage kidney disease (ESKD). Traditional kidney damage assessment methods are semi-quantitative and subjective. This study aims to use a convolutional neural network (CNN) to segment murine kidney structures after IRI, quantify damage via CNN-generated pathological measurements, and compare this to conventional scoring. The CNN was able to accurately segment the different pathological classes, such as Intratubular casts and Tubular necrosis, with an F1 score of over 0.75. Some classes, such as Glomeruli and Proximal tubules, had even higher statistical values with F1 scores over 0.90. The scoring generated based on the segmentation approach statistically correlated with the semiquantitative assessment (Spearman’s rank correlation coefficient=0.94). The heatmap approach localised the intratubular necrosis mainly in the outer stripe of the outer medulla, while the tubular casts were also present in more superficial or deeper portions of the cortex and medullary areas. This study presents a CNN model capable of segmenting multiple classes of interest, including acute IRI-specific pathological changes, in a whole mouse kidney section and can provide insights into the distribution of pathological classes within the whole mouse kidney section.

## Teaser

**Summary:** This study employs AI-driven analysis of kidney tissue slides, enhancing the understanding of disease progression using mouse models. Deep learning's potential to improve injury assessment methods is explored, challenging conventional standards.

## INTRODUCTION

Acute kidney injury (AKI) is a global public health problem, with rising incidence and mortality rates in recent decades (Lameire et al., 2013). AKI is caused by various pathological processes, including ischemia-reperfusion injury (IRI), follow-on effects of kidney transplantation, drug toxicity, sepsis, and other insults (Lameire et al., 2013). AKI resulting from IRI has complex pathogenesis, and experimental animal models of kidney IRI offer the possibility to examine key pathogenesis-related morphological changes. The gold standard to assess kidney injury remains semi-quantitative histopathology scoring; however, traditional scoring systems are time consuming, in some cases subject to inter-observer variability and are based only on a small sample area of the kidney section (Wang et al., 2005), hence lacking quantitative power. In addition, kidney tissue has a complex non-redundant architecture, rendering semi-quantitative scoring intrinsically challenging due to the potential lack of representativeness of randomly selected areas of interest. Moreover, the scoring systems available differ in terms of morphological structures and areas analysed (Hesketh et al., 2014; Wang et al., 2005), and a comparative analysis of their performance is lacking.

Digital image analysis techniques can improve the visual assessment of a wide range of images (Kshirsagar and Joshi, n.d.), including histological microphotographs (Niazi et al., 2019). The advantages of using computer algorithms include increased reproducibility and, with sufficient computational power, the ability to analyse the whole sample or multiple batches of samples in automation. As visual assessment is monotonous and prone to lack of objectivity, it represents a significant limiting factor in extensive studies, and the use of automated assessment could overcome this. The traditional approach to performing image analysis refers to computer vision methods such as feature descriptors for object detection (O'Mahony et al., 2020). For tasks such as image classification, a feature extraction step is needed. The main inconvenience with this method is that it is necessary to choose *a priori*, which features are valuable in each image. Therefore, as the number of classes increases, this step becomes burdensome. These methods have been successfully used in small kidney histopathology studies; one study developed a segmental histogram of oriented gradients that successfully performed a comprehensive detection of glomeruli in whole kidney sections (Kato et al., 2015) while another study (Grimm et al., 2003) used computerised image analysis of Picro Sirius Red-stained kidney tissue sections to quantify the extent of collagen and consequently interstitial fibrosis, a valuable predictor of long-term graft function. Although these traditional image analysis techniques can be useful to answer specific research questions, it would be challenging to automatically apply them in large-scale studies with data sets that are likely to present variations. Moreover, it is up to the computer vision scientist to decide which features best define the various classes of objects after a time-consuming trial-and-error approach. On top of that, a plethora of parameters are required to define each feature, all of them having to be fine-tuned by the operator (O'Mahony et al., 2020). Finally, in some cases, traditional machine-learning approaches may not detect complex morphological features.

In the last 2 decades, digital imaging has seen the emergence and progress of whole slide imagining (WSI), which permits full slides to be digitised and stored at high resolution (Niazi et al., 2019). More recently, with the advent of the graphics processing unit (GPU)-based computation and convolutional neural networks (CNNs), a full histological section can be completely and consistently analysed for several objects of interest, using a supervised training strategy without the need to pre-set the features for each object. CNNs represent a deep-learning method that draws inspiration from the intricate organisation of human brains. By employing a model structure comprising multiple processing layers, deep learning (DL) allows for the acquisition of diverse levels of data representation, leading to unprecedented improvements in model performance. This state-of-the-art technology has revolutionised various fields, including speech recognition, visual object identification, and drug discovery and genomics domains (Xie et al., 2020). CNNs are the most applied deep-learning models for bio-image analysis. CNNs mimic the human visual perception process via a cascade of interconnected, layered units (neurons) that resemble the visual system architecture (Lindsay, 2021; Tang et al., 2019). In contrast with the traditional approaches for image analysis, neural networks can be trained to automatically detect underlying patterns in classes of images that have been previously labelled and extract and the most descriptive features (O'Mahony et al., 2020). Previous work involving CNNs applied to WSIs focused on classifying human cancers, such as invasive breast cancer (Cruz-Roa et al., 2017) and identifying pancreatic endocrine tumours (Niazi et al., 2018), among others. In animal models, CNNs have also been applied to histologically score lung fibrosis and inflammation (Heinemann et al., 2018) and to assess the severity of various pulmonary lesions (Asay et al., 2020). The use of CNNs in the domain of kidney histopathology is a relatively recent development, and the existing body of literature primarily focuses on the detection of glomeruli (Bukowy et al., 2018; Gallego et al., 2018). The latest investigations pertaining to human kidney biopsies have also directed their attention towards the segmentation of multiple classes of interest within distinct scenarios. For instance, some studies have specifically targeted the segmentation of classes within healthy kidney tissue (Marechal et al., 2022), while others have concentrated on biopsies associated with IgA-nephropathy (Hölscher et al., 2023) and chronic kidney injury (Ginley et al., 2021). Furthermore, certain studies have exclusively addressed the segmentation of the healthy kidney cortex or both the cortex and medulla, omitting the inclusion of vital components such as the papilla, transitional epithelium, and associated connective tissue (e.g. stroma and adipose tissue), which frequently appear in the observed sections (Bukowy et al., 2018; Hermsen et al., 2019). However, no study so far has included the CNN-based segmentation on a specific disease model quantifying both injured and healthy structures on an entire mouse kidney section. The present study aimed at applying CNNs to kidney WSI and testing their efficacy to detect levels of kidney damage in an IRI mouse model using a segmentation approach and to compare it to a widely used traditional semi-quantitative method.

## RESULTS

### CNN multiclass segmentation performance

**Fig. 1.**
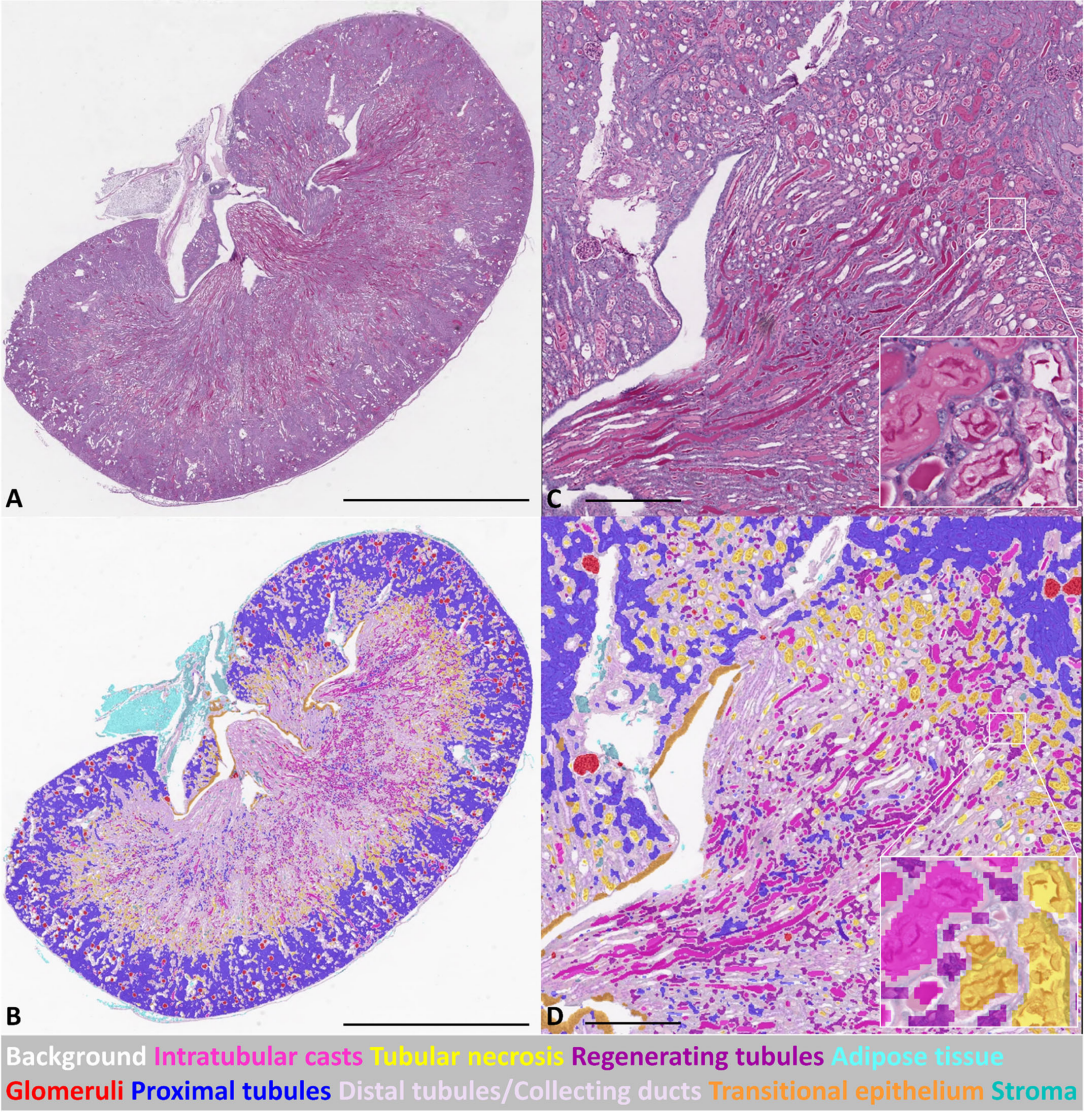
**CNN-based automated segmentation on WSI of an injured murine kidney on day 3 after IRI.** (A) PAS stained WSI; (B) corresponding segmentation result of WSI in A. (C) High-magnification PAS stained WSI, with further higher magnification area (inset); (D) corresponding segmentation result of WSI in C, with further higher magnification area (inset). Scale bars: 2 mm (A,B); 400 um (micrometers) (C,D).

Multiclass semantic segmentation of kidney sections enabled the extraction of quantitative histological features on a large scale. An example of a fully segmented pathological mouse kidney section is depicted in Fig. 1 (a healthy segmented kidney is presented in Fig. S1).

**Fig. 2.**
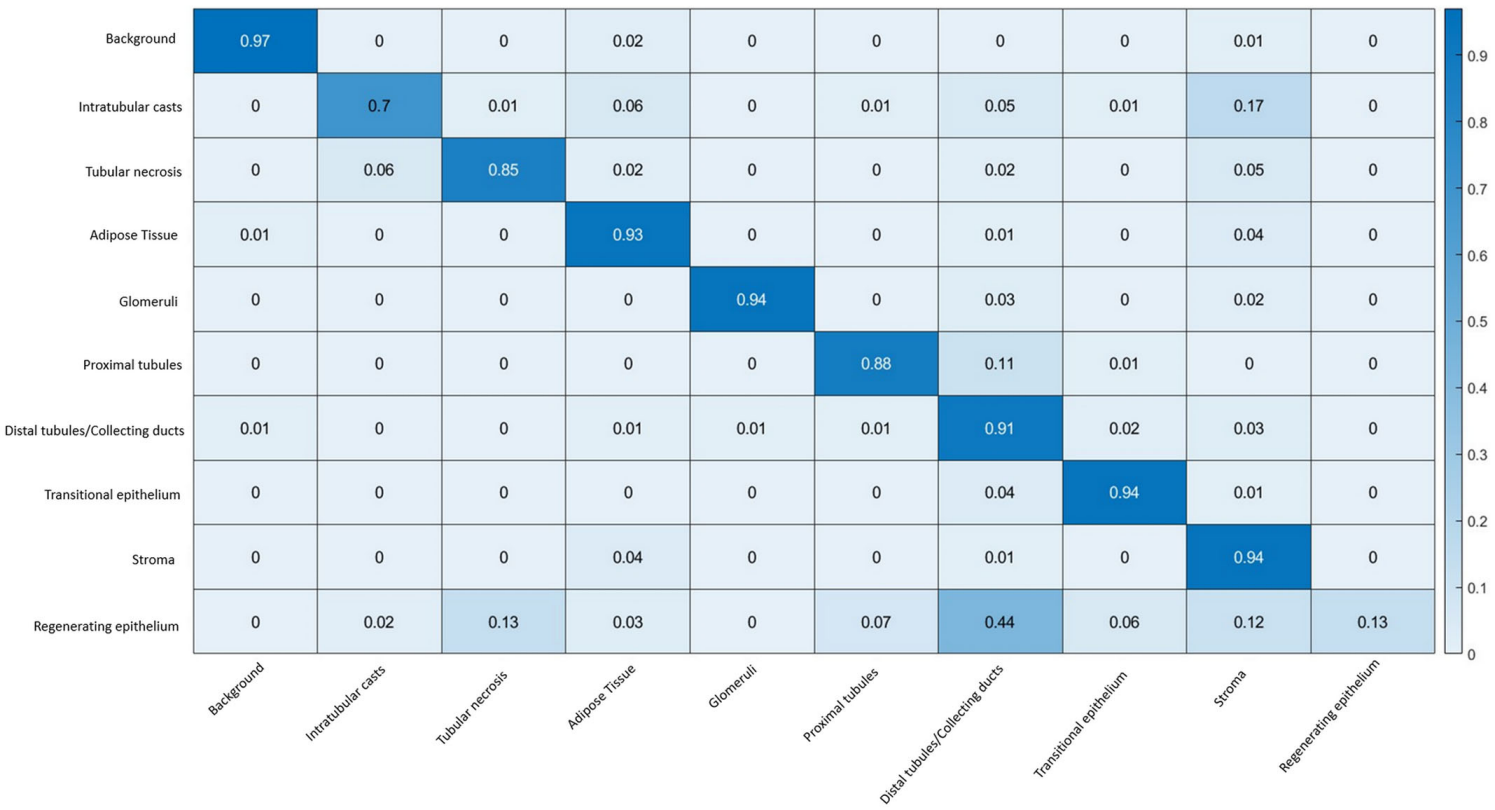
**Heat map confusion matrix for the CNN performance on the test set of mouse kidney IRI sections.** The ground truth classes are given vertically (percentage of pixels), and the predicted classes (percentage of pixels) are shown on the horizontal axis. Examples of readings: 85% of all pixels labelled as ‘Tubular necrosis’ were classified as ‘Tubular necrosis’ by the DL model; 94% of all pixels labelled as ‘Glomeruli’ were classified as ‘Glomeruli’ by the DL model; 44% of the pixels labelled as ‘Regenerating epithelium’ were misclassified with the ‘Distal tubules/Collecting ducts’ class.

The segmentation performance on the test set was assessed via a confusion matrix (Fig. 2) from where precision, recall, specificity and F1 were extracted (Table 1). Good performances were obtained for the ‘Glomeruli’ class, where 94% of the ground truth labels were correctly identified with a precision of 0.99. Segmentation performances for ‘Stroma’, ‘Transitional epithelium’, and ‘Adipose tissue’ were similar to the ‘Glomeruli’ class (above 90%) but with lower precision. For the ‘Proximal tubule’ class, 88% of all pixels labelled with this name were correctly classified by CNN with a precision >0.95. As regards the classes representing pathological changes, ‘Intratubular casts’ and ‘Tubular necrosis’, 70% and 85% of the ground truth pixels were correctly classified, respectively, with a precision of 0.87 and 0.94. The overall model statistics are presented in Table S3.

**Table 1.**
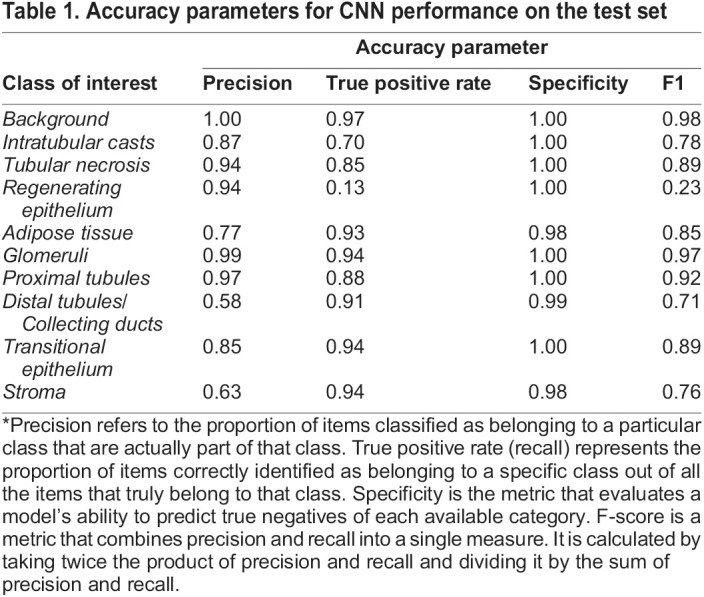
Accuracy parameters for CNN performance on the test set

Lower precision values were observed for the ‘Regenerating epithelium’ class, and the misclassification was mainly with the ‘Distal tubules/Collecting ducts’ class (44%). The ‘Regenerating epithelium’ class was further excluded from the generation of the CNN-based scoring due to its lower statistical values.

### CNN-based IRI scoring versus semi-quantitative IRI scoring method and heatmap

**Fig. 3.**
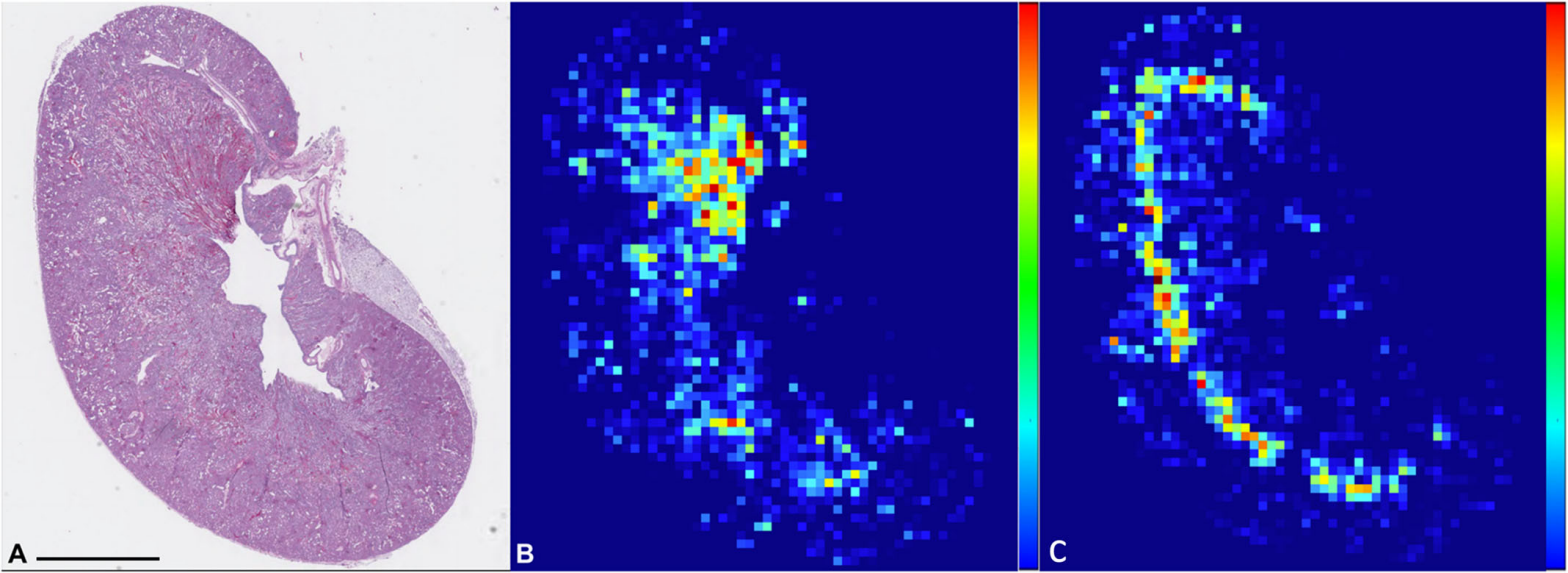
**PAS stained WSI of mouse kidney (A) and heatmaps of ‘Intratubular casts’ (B) and ‘Tubular necrosis’ (C).** Percentage of area of each patch (512×512 pixels) occupied by pathological classes is represented as colours ranging from deep blue (0%) to red (100%) in order to spatially visualise the pathological classes within kidney parenchyma. Scale bars: 2 mm.

Two classes (Intratubular casts and Tubular necrosis) were selected as representative of pathological changes as they presented good statistical values (F1=0.78 for Intratubular casts class and F1=0.89 for Tubular necrosis class). The network applicability to score IRI damage was assessed by comparing CNN's quantification of selected classes, ‘Tubular Necrosis’ and ‘Intratubular casts’ (Table S2), to the semi-quantitative scoring method. Upon heatmap analysis (Fig. 3), the classes Intratubular casts and Tubular necrosis were clearly spatially identified. Areas of more intense necrosis were often focused in between the cortex and medulla (Outer stripe of outer medulla; OSOM), a region known to be affected by hypoxic damage (2,3). Casts were often associated with the same area but varied in localisation, involving more superficial or deeper portions of the cortex and medullary areas.

**Fig. 4.**
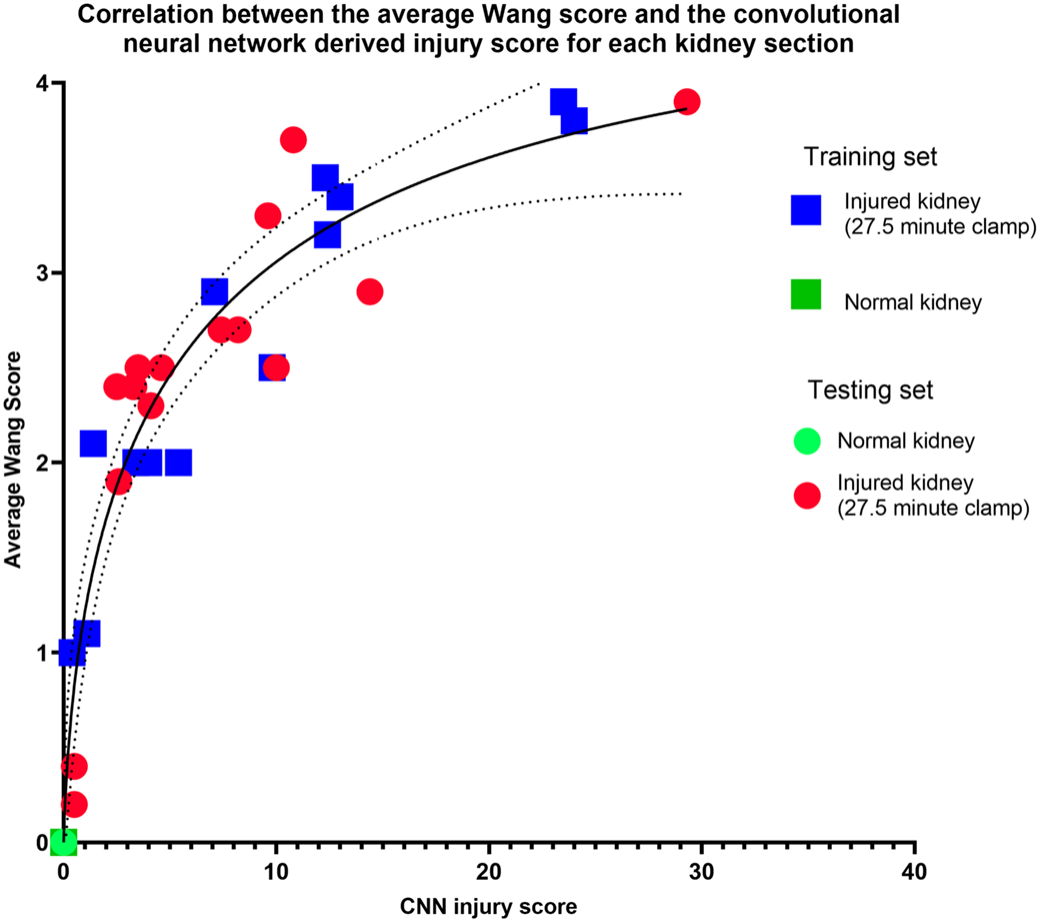
**Scatterplots visualising the correlation between the traditional scoring systems and CNN-based scoring per kidney section (*N*=34; Normal kidney results overlap at 0).** The conventional method of scoring appears highly correlated to the CNN scoring, with the Spearman Correlation coefficient=0.94 (*P*<0.0001).

The conventional semiquantitative method of scoring appeared highly correlated to the CNN scoring with a Spearman’s rank correlation coefficient (31)=0.94 (*P*<0.0001) (Fig. 4).

## DISCUSSION

Acute IRI models are, at present, evaluated morphologically using semi-quantitative histological methods. Limitations of this approach include lack of reproducibility, interobserver variability and limited sample analysis. Additionally, the systems used may differ in terms of the morphological structures and areas that are analysed (Hesketh et al., 2014; Sun et al., 2016; Wang et al., 2005). Here, we have developed a novel system to quantify kidney damage in a mouse model of IRI by using a DL approach. Compared to the traditional way of scoring IRI, where only ten fields of view focused on the OSOM are being used, which represents approximately 10% of the kidney surface, our methods use WSIs that allow a full assessment of the mouse kidney section, increasing the quantitative power of the scoring method. We have obtained good segmentation results for the majority of classes of interest, with 9/10 presenting an F1 value higher than 0.70.

With an overall model precision of 0.85, these results align with previous work on human kidneys (Hermsen et al., 2019). The present study represents the first deep learning model to simultaneously segment and classify nine classes of interest on a full mouse kidney section originating from ischaemia-reperfusion injury surgery. Previous work focused only on detecting glomeruli (Gadermayr et al., 2019) in mouse sections or creating multiple networks for different morphological structures in human samples (Jayapandian et al., 2021), possibly increasing the time required to segment and classify all structures. Using a single network to identify multiple structures accurately decreases the overall time needed for full classification. The DL model described here was trained and tested on single stain (PAS) WSIs, compared to Jayapandian and colleagues (Jayapandian et al., 2021), who tested a CNN approach on multiple stains (Hematoxylin and Eosin, H&E, PAS, Silver, and Trichrome). This also represents an advantage of our system in terms of time and material needed for the analysis. The selection of PAS staining was based on its ability to provide a more precise evaluation of the basement membrane when compared to H&E staining. Furthermore, PAS staining was preferred due to the increased positivity exhibited by the proteinaceous casts in contrast to H&E staining, where the casts appear significantly lighter (Dvanajscak et al., 2020). This study used pathological sections from three IRI experiments, with the experimental design being consistent with one another but conducted at different time points. A fourth experiment had a distinct experimental setup, featuring unilateral rather than bilateral ischemia and a longer duration. This study used only the unclamped kidneys from the unilateral IRI experiment as healthy controls. Additionally, sections were processed in two separate laboratories to account for domain shift, encompassing variations in experimental conditions and processing techniques such as embedding. It is important to note that the PAS staining was performed in the same laboratory, thereby limiting the debate on staining variability in this study. However, colour normalisation can be used in cases where staining intensity varies. Moreover, the DL model performed well on both healthy and pathological sections. The best segmentation performance was achieved for the ‘Glomeruli’ class (F1>0.95), as similarly achieved in previous studies on the human kidney (Hermsen et al., 2019).

The DL model also demonstrated great potential to distinguish between proximal (F1>0.90) and ‘Distal tubules/Collecting ducts’ (F1>0.70). Although the focus of the work was to achieve a system to quantify pathological classes, the identification of multiple healthy classes compared to a single ‘healthy kidney tissue’ class opens the option of more detailed types of analyses, such as the quantification of the amount of damage per number of glomeruli, per nephron or tubular area. In addition, this approach allows spatial analysis (e.g. the average distance between specific healthy areas and pathological ones) between different classes using a heatmap approach which can complement the quantitative analysis of the whole section.

In histopathology, overfitting can occur when the DL model has learned to recognise the unique features of the training images set, but it does not generalise well to new images that contain different variations of the same tissue structure. In generating the CNN injury score, we deemed it appropriate to extract quantification results from both our training and testing datasets, as overfitting does not represent a crucial issue when the task is to accurately quantify an experimental dataset of WSIs from which annotation examples will be generated. In this regard, each WSI offers training and testing areas within the same slide. Two pathological classes, namely ‘Intratubular casts’ and ‘Tubular necrosis’, were considered reliable and were further used to score damage in the mouse kidney sections. These two classes represent the most widely used parameters in different scoring systems to identify acute IRI changes (Hesketh et al., 2014; Sun et al., 2016; Wang et al., 2005). Necrosis, in particular, overlapped as expected with OSOM in most samples, as mentioned in previous studies (Hesketh et al., 2014; Wang et al., 2005).

‘Regenerating epithelium’ represented the least successful class to segment efficiently. The regeneration process in the kidney is a complex process encompassing a continuum of pathogenetic transition (degeneration, necrosis, regeneration) and associated different morphological hallmarks (e.g. flattened cells, mitotic figures, and plump cells). Therefore, obtaining the ground truth for this class presented as quite challenging, likely due to the intrinsic variable morphology of the class itself overlapping with a single concept of ‘regeneration’. This translated into minor statistical values (F1>0.20) and would benefit from more training data or a further splint into the morphological stages of this particular change, which is beyond the scope of the present study. In addition, because the mice in the acute IRI model were only kept for 3 days after surgical induction of IRI, the regenerating process was minimally represented compared to the necrotic phase identified, suggesting that this class should be likely better characterised in subsequent timeframes of the IRI and regeneration process (e.g. sub-acute, chronic) (Frazier et al., 2012). It is important to note that the ‘Regenerating epithelium’ class represented a very small portion of the segmented kidney area (average 0.02%) and was confused mainly with the ‘Distal tubules/Collecting ducts’ class, not interfering significantly with the classes we used to score IRI damage. Pitfalls related to morphologically difficult classes are expected in this type of study, as seems to be the case in the work of Hermsen and colleagues (Hermsen et al., 2019), where the class ‘empty bowman capsule’ is likely overlapping large veins according to the segmentation masks provided.

The CNN-based scoring was compared to a previously used semiquantitative way of scoring performed by a board-certified pathologist. The results support a positive correlation between the pathologist and the DL model for detecting IRI severity in an acute mouse model, suggesting that the algorithm correctly assesses general features that are accepted signs of IRI by pathologists. As a general trend, the DL algorithm assigned lower grades than the pathologist. This could be because the area analysed has been dramatically extended, and/or the traditional way of scoring looks only at the OSOM, a region known to be affected by hypoxia (Hesketh et al., 2014; Wang et al., 2005), ignoring all the other kidney regions. In addition, it is important to consider that the pathologist's personal perception may unintentionally lead to the selection of more severely injured areas for analysis. However, the pathologist and the CNN scorings overlap in the healthy non-injured kidney sections (*n*=3) as both assigned a score of 0. It is important to note that the inter-observer variability issue was not addressed in this study, as the quantification by the CNN was only compared to the opinion of one pathologist.

Heatmaps were drawn based on the quantification of ‘Intratubular casts’ and ‘Tubular necrosis’ of each grid patch. As expected, most of the damage was localised in the OSOM region of the kidney (Hesketh et al., 2014; Sun et al., 2016; Wang et al., 2005). However, in several cases, a substantial amount of damage (mainly cast formation) was visually identified in cortical and medullary areas, indicating that traditional scoring systems that analyse fields of view only from the cortex or only from the OSOM might not provide the most accurate assessment.

Many studies developed DL models to detect and grade tumours in the past decade. In kidney histopathology, the morphological changes associated with disease (e.g. IRI, allograft rejection) are more complex in appearance compared to the more spatially homogeneous morphological landmarks of the neoplastic process; from this point of view, our approach represents a useful DL algorithm for the segmentation and classification of renal structures that is applicable to the preclinical field of IRI.

This study presents a CNN model capable of segmenting and classifying multiple classes of interest, including acute IRI-specific pathological changes, in a whole mouse kidney section. Moreover, the DL model was applied to sections from different IRI experiments, suggesting that the model generalises well and can represent a useful tool for quantitatively investigating acute IRI models upon histology.

## MATERIALS AND METHODS

### Animals and surgery

All experiments were conducted in accordance with the Animals (Scientific Procedures) Act 1986 under a project licence (PPL 7008741 and PP3076489) and were approved by the Animal Welfare and Ethical Review Board (AWERB) of the University of Liverpool. B6 albino mice (C57BL/6J.Tyrc-2J) were purchased from Charles River, Italy, and used to establish a colony that was maintained by the Biomedical Services Unit (BSU) at the University of Liverpool, UK. Mice were housed in ventilated cages with a 12-h light:dark cycle and access to water and food *ad libitum*.

The mouse model of renal IRI was induced by bilateral clamping of the renal pedicle using a dorsal approach. Male mice (9–10weeks) were anaesthetised (isoflurane 1.5%; 1.0 L/min, O_2_) for 30 min prior to the surgical procedure (Harwood et al., 2019). The body temperature was controlled at 36.5–37.1°C with a homeothermic monitor system (PhysioSuite, Kent Scientific, Torrington), and the renal pedicle was carefully dissected and clamped with a non-traumatic vascular clamp (InterFocus Ltd, Liton, 18052-03) for 27.5 min. The confirmation of ischaemia was observed through a colour change in the kidney, from red to dark purple. Once the clamp was released, the kidney returned to its normal red colour, indicating reperfusion. Subsequently, the surgical wound was repaired, and the animal allowed to recover in a warmed chamber at 37°C for 30 min prior to returning to their cage.

Four different sets of experiments were carried out, three of them following the procedure described above. The fourth experiment followed the same surgery and anaesthesia methods, except the clamp was unilateral right-sided and for a period of 40 min. Only the unclamped kidneys from the unilateral IRI experiment were used from this fourth study as healthy controls.

Animals were euthanized via cervical dislocation 3 days post-IRI surgery, kidneys exteriorised after laparotomy and immediately fixed in 10% neutral buffered formalin (Thermo Fisher Scientific, Leicestershire, 10463750) for 24–72 h.

### Histology and semi-quantitative scoring

Mice kidneys were fixed in 10% neutral buffered formalin (Thermo Fisher Scientific, Leicestershire,10463750) for 24–72 h. After fixation, kidneys were sagittally sectioned in two halves and placed in formalin into slotted tissue cassettes (Thermo Fisher Scientific, Leicestershire, 15327260) until further processing (Morawietz et al., 2004). The specimens were paraffin–embedded and sectioned, using standard procedures, by the LBIH Biobank (University of Liverpool, Liverpool, UK) or the Veterinary Pathology Laboratory, Department of Veterinary Anatomy and Physiology. Sections of 3–4μm were mounted on glass slides, stained with Periodic Acid-Schiff (PAS) and coverslipped by the Veterinary Pathology Laboratory (Leahurst Campus, University of Liverpool, Wirral, UK) and dried for histological analysis.

A total of 34 mid-coronal renal full sections, originating from 18 animals, were used in the study. These included 28 sections originating from 15 animals (23 clamped kidneys), subject to direct IRI and six sections originating from three animals (unclamped kidneys).

Subsequently, the slides were scored using a semi-quantitative scoring as previously performed by the same group (Sharkey et al., 2019), adapting a method previously described by Wang and colleagues (2005). Briefly, kidney lesions were scored depending on the displayed pathological changes (tubules that displayed typical changes of the IRI model, including cell necrosis, intratubular cast formation, reduction of brush border, tubular dilation and tubule regeneration) on ten randomly selected fields of view (FOV) from the outer stripe of the outer medulla (OSOM) and cortex. The FOVs were given a score of 0–4 where 0=0%; 1=1–25%; 2=26–59%; 3=51–75%; 4=76%–100%, then the average of 10 FOVs scores were considered the final score for each tissue section. Scorings were performed using a brightfield microscope (Leica Biosystems, Nussloch, Germany) at 200X magnification by a board-certified veterinary pathologist (LR).

### Digitisation and neural network training and analysis

PAS-stained slides were digitally scanned using the Aperio CS2 slide scanner (Leica Biosystems, Nussloch, Germany), with Plan Apo 20X objective lens setup, image size ranging from 21,000 to 35,000-pixel width and 13,000-to-31,000-pixel height (0.504 microns per pixel), and visualised using ImageScope™ software (Leica Biosystems, Nussloch, Germany).

The WSIs (*n*=34) were randomly split into training and testing sets as follows: 17 training and 17 testing. The ground truth was created by manually annotating regions corresponding to normal or pathological changes: of the following normal renal structures represented classes as per normal microscopic anatomy: ‘background’ (area of the slide characterised by the homogeneous white area without the presence of any histological structure), ‘adipose tissue’ (area of the stroma characterised by large numbers of adipocytes), ‘glomeruli’ (round structures that represent a complex web of capillaries), ‘proximal tubules’ (elongated structures with abundant, pink cytoplasm and an easily identifiable brush border), ‘Distal tubules and collecting ducts’ (tubular structures with wider lumen, no brush border and less pink cytoplasm than proximal tubules in the cortex and tubules within the medulla), ‘stroma’ (connective tissue containing fibroblasts) and ‘transitional epithelium’ (multiple cuboidal layers of epithelium within the renal pelvis). Pathological classes selected for the purpose of model training were: ‘intratubular casts’ (uniformly staining proteinaceous material structures found within/filling the tubular lumen): ‘tubular necrosis’ (destruction of tubular epithelial cells shedding into the tubule lumen): ‘regenerating epithelium’ (tubular epithelium with flattened cells, and/or more cuboidal cells and/or mitotic figures). References (Percy and Barthold, 2007; Scudamore, n.d.) were used during annotations of normal and pathological structures. Annotations were performed by one investigator (AL) and reviewed by a board-certified veterinary pathologist (LR). An example of annotated tissue is available in Fig. S1. The ‘intratubular casts’, ‘tubular necrosis’, and ‘regenerating epithelium’ classes were considered representative of pathological changes associated with IRI. Cutting/staining artefacts were rarely present on the slides and were not included in the annotations. The number of annotations was automatically balanced to a median of 288 per class. The total number of training annotations used to train the CNN model was 2880 in total (Table S1).

The deep-learning process took approximately 8 days on a system equipped with 4x Nvidia^®^ Quadro^®^ RTX8000 GPUs (Nvidia, Santa Clara, CA, USA) using dedicated software MIMPro (Medical Image Manager Pro with Deep Learning Add On; HeteroGenius^®^). The employed CNN model is composed of the descending, downsampling portion of a UNET architecture (Ronneberger et al., 2015), where upsampling layers were not included with an 8× downsampled mask as an output. The network was trained for a total of 2300 epochs on the training set, at one iteration per epoch, with batch sizes of 1, 32 and 64. Patches of 512×1024 pixels and magnification of 20× (18,167×17,001 pixels) were used. Adaptive learning rates between 5e-7 and 5e-4 and momentum with values of 0.9 and 0.99 were used depending on monitoring error curve progress ‘on the fly’. Data balancing, dropout (ranging from 0 to 0.1) and random transforms were used to increase generalisation and improve the algorithm's robustness for variation in tissue section morphology and staining intensity.

The model obtained was deployed through MIMPro^®^ to create a segmentation where individual pixels are assigned to one of the pre-defined classes. Subsequently, an overlay image (mask) was created where each classified pixel was assigned a colour relating to its class, making the quantification of the pre-defined classes possible. The model was then tested on the test set.

### Application of CNN to score acute IRI lesions

All classified areas in pixel except ‘background’ were summed together for each WSI, representing the surface of the kidney section. The number of pixels representing the pathological classes was transformed into percentages as follows:

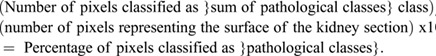
The percentage of pixels classified as ‘pathological classes’ was considered the score assigned by the CNN model.

To visually map the quantified pathological areas of each single pathological class within the processed WSI, a grid of patches was created and overlayed on the digitally scanned slides (MIMPro^®^). For each patch of 512×512 pixels, the area covered by the pathological classes was quantified using the previously developed CNN and expressed as a total number of pixels belonging to the class of interest per patch. Pathological classes were then spatially visualised within the kidney parenchyma using a heat map where in a gradient, low values of the classes of interest are represented in blue and high values in red.

### Data analysis

To summarise the algorithm's performance, a multiclass confusion matrix was created, from where precision (fraction of predictions as true positives), recall (sensitivity), specificity and F1 (harmonic mean between precision and recall) values were extracted. After the true positives (TPs), true negatives (TNs), false positives (FPs), and false negatives (FNs) were estimated using a confusion matrix, precision, recall and specificity were calculated using the following formulas:

Precision=TP/(TP+FP); Recall=TP/(TP+FN)]; Specificity=TN/(TN+FP).

The F1 was calculated using the following:

2×PrecisionxRecall/(Precision+Recall)=2TP/(2TP+FP+FN)

Pearson's correlation coefficients were calculated among the traditional Wang and CNN-based scoring on both datasets (training and testing). Significance was set as *P*<0.05.

## Supplementary Material

10.1242/biolopen.059988_sup1 Supplementary information
