# Supplementary material for: Quantifying acute kidney injury in an Ischaemia-Reperfusion Injury mouse model using deep-learning-based semantic segmentation in histology
Source: Biol Open. 2023 Sep 21;12(9):bio059988. doi: 10.1242/bio.059988 (PMC10537956; doi:10.1242/bio.059988)
Supplement: Supplementary information [file biolopen-12-059988-s1.pdf]

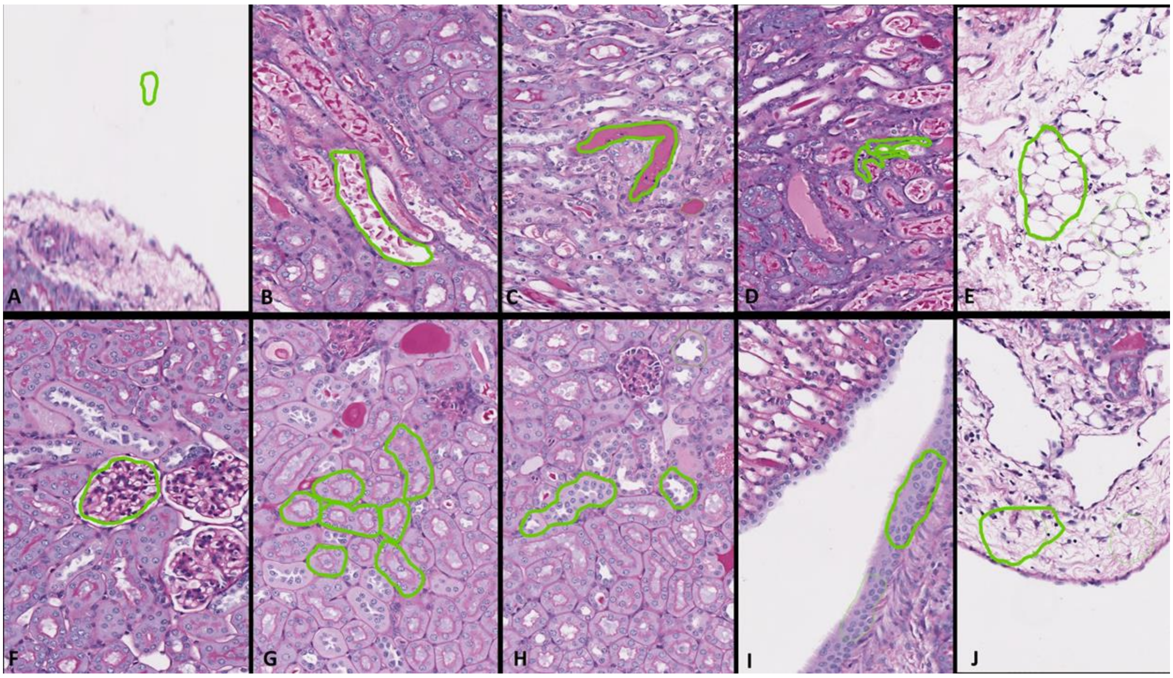

Fig. S1. Annotation procedure: A – Background, B – Tubular necrosis, C – Intratubular casts, D – Regenerating epithelium, E – Adipose tissue, F – Glomeruli, G – Proximal tubules, H – Distal tubules, I – Transitional epithelium, J – Stroma

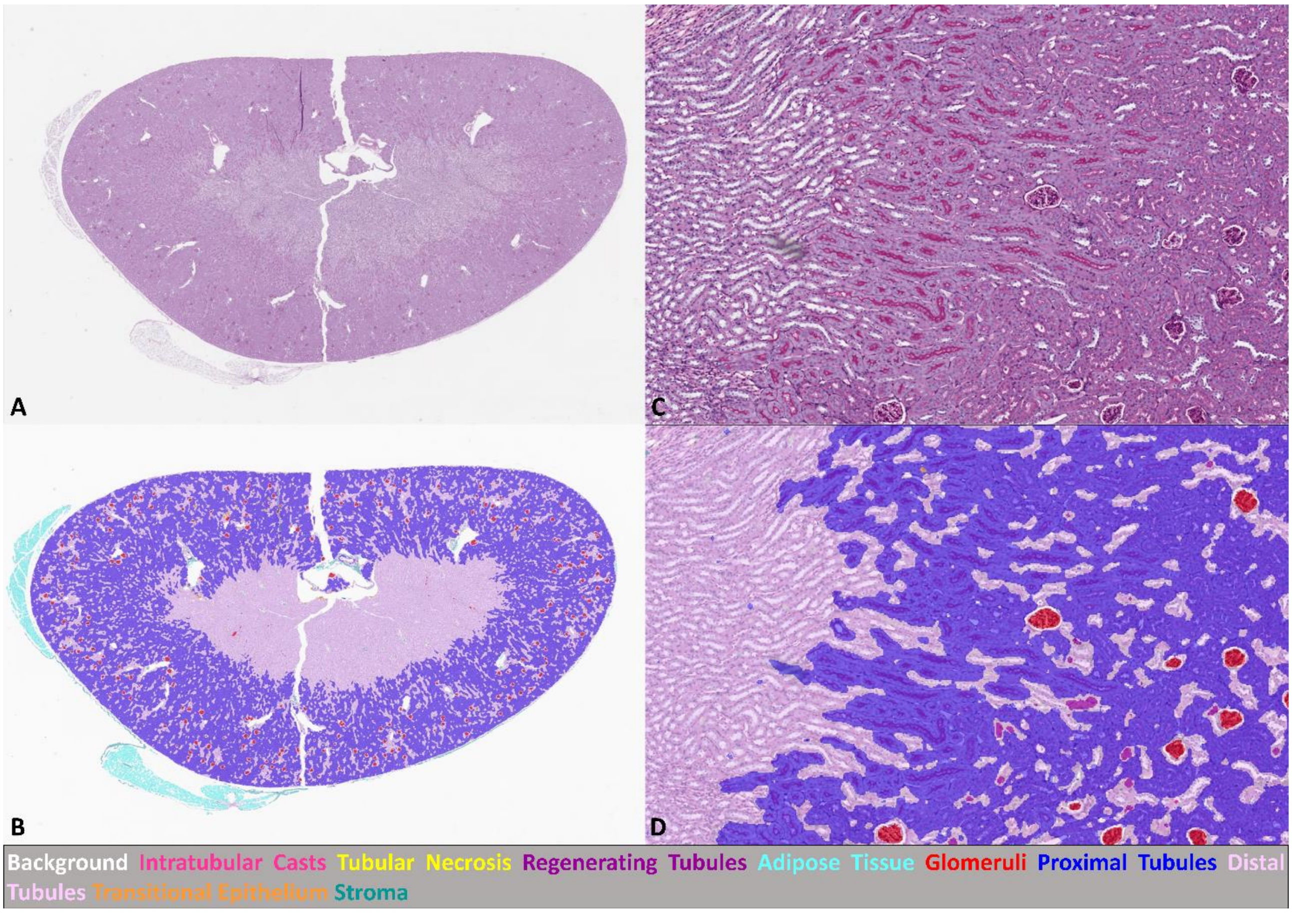

Fig. S2. Automated segmentation on WSI of a healthy murine kidney. (A) - full PAS stained WSI and its corresponding segmentation result (B); (C)- High magnification PAS stained WSI and its corresponding segmentation result (D).

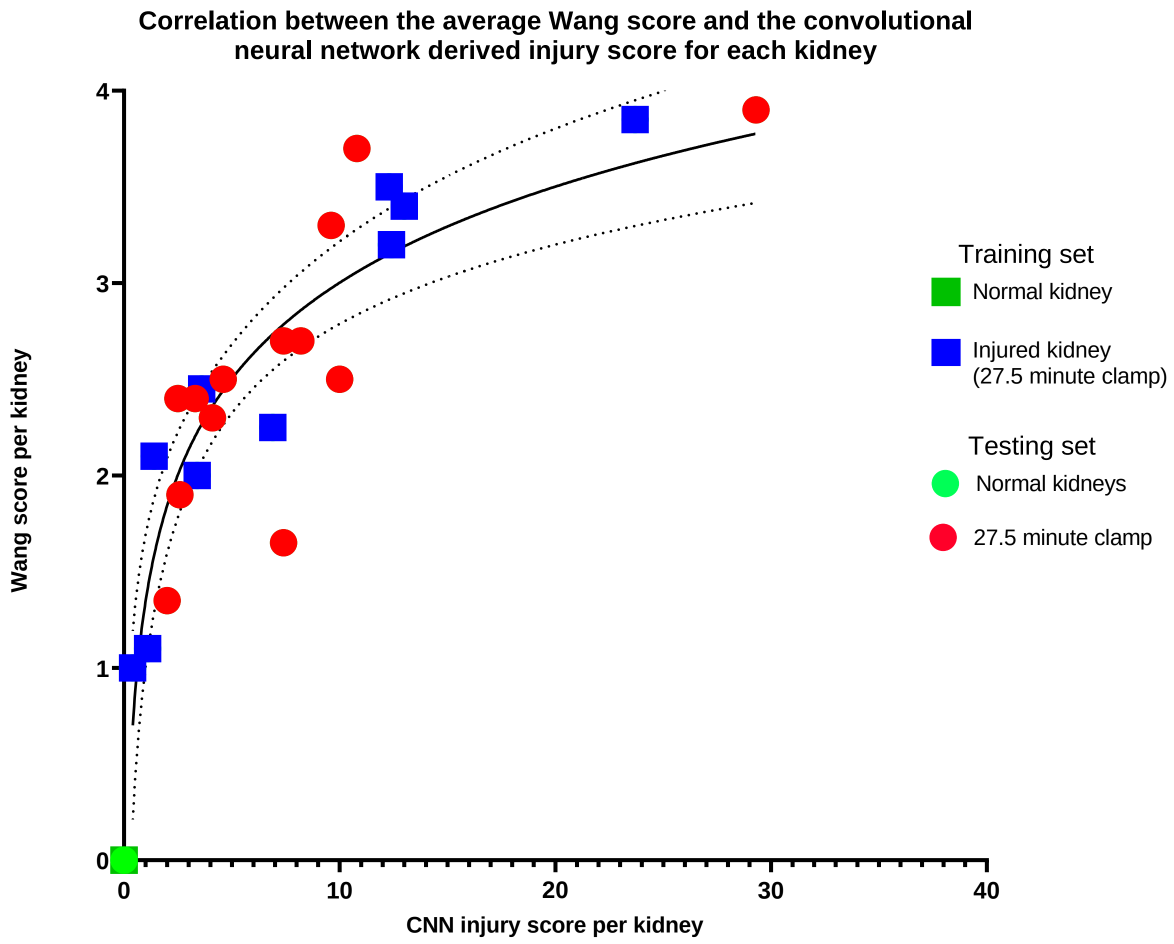

Fig. S3. Scatterplots visualising the correlation between the traditional scoring systems and CNN-based scoring per kidney. The conventional scoring method appears highly correlated to the CNN scoring, with the Spearman Correlation coefficient = 0.92 ($p<0.0001$).

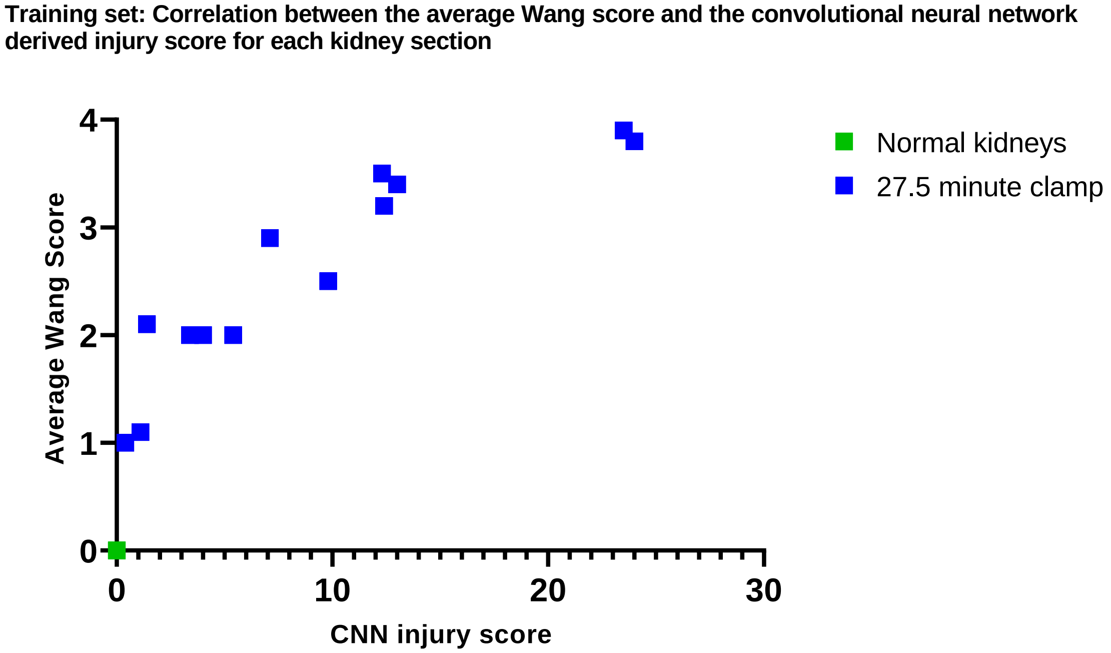

Fig. S4. Scatterplots visualising the correlation between the traditional scoring systems and CNN-based scoring per kidney section in the training set. The conventional scoring method appears highly correlated to the CNN scoring, with the Spearman Correlation coefficient = 0.95 (p<0.0001).

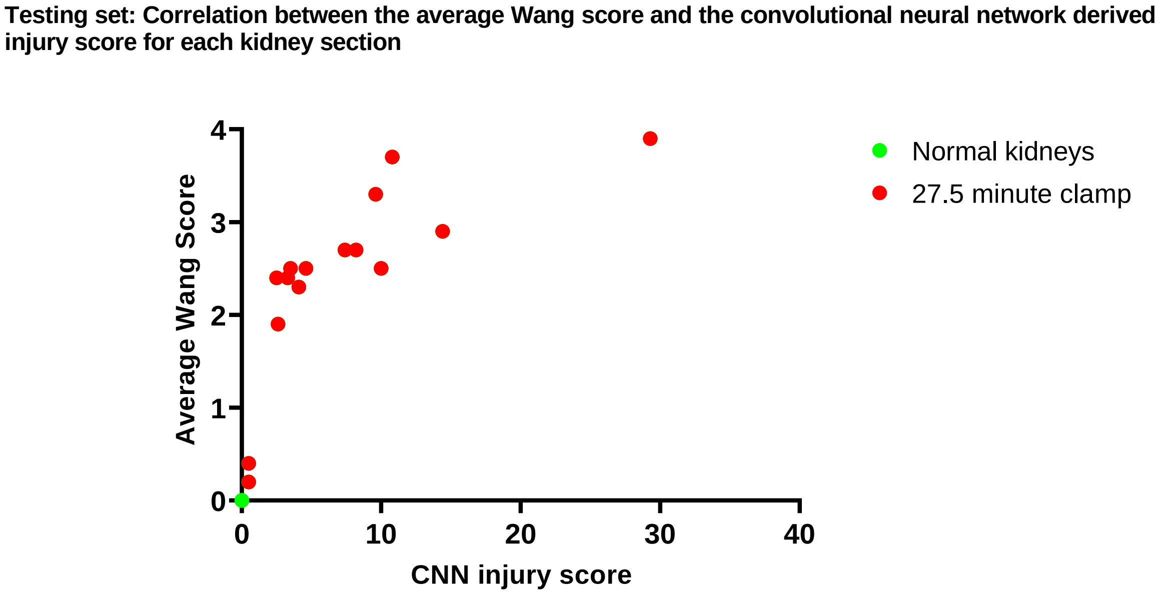

Fig. S5. Scatterplots visualising the correlation between the traditional scoring systems and CNN-based scoring per kidney section in the testing set. The conventional scoring method appears highly correlated to the CNN scoring, with the Spearman Correlation coefficient = 0.92 (p<0.0001).

Biology Open • Supplementary information

Table S1. Quantitative information on ground truth data

Class	Number of annotations	Number of pixels
Background	288	76952097
Intratubular casts	288	2198198
Tubular necrosis	288	3033026
Regenerating epithelium	288	8459979
Adipose tissue	288	3248413
Glomeruli	288	3871080
Proximal tubules	288	3579512
Distal tubules/Collecting ducts	288	3858445
Transitional epithelium	288	8275538
Stroma	288	888184

Table S2. IRI damage scorings (test + train) performed by the DL model and the pathologist

Case	DL (CNN score)	Pathologist score (Wang modified)
1	1.10%	1.1
2	4.00%	2
3	9.80%	2.5
4	5.40%	2

Biology Open • Supplementary information

5	7.10%	2.9
6	23.50%	3.9
7	24.00%	3.8
8	13.00%	3.4
9	12.40%	3.2
10	3.40%	2
11	0.40%	1
12	1.40%	2.1
13*	0.00%	0
14*	0.00%	0
15	12.30%	3.5
16	10.00%	2.5
17	0.50%	0.4
18	14.40%	2.9

Biology Open • Supplementary information

19	0.50%	0.2
20	3.50%	2.5
21	2.50%	2.4
22	4.60%	2.5
23	8.20%	2.7
24	2.60%	1.9
25	7.40%	2.7
26	9.60%	3.3
27	3.30%	2.4
28	10.80%	3.7
29	29.30%	3.9
30	4.10%	2.3
31*	0.00%	0

Cases marked with an * - 2 sections per slide, scores represent the average of those 2 consecutive sections

Table S3. Overall model statistics

Mean Precision	0.85
Mean True Positive Rate	0.82
Mean Specificity	1
Mean F1	0.80

Table S4. Accuracy parameters for CNN performance on the training set

	Accuracy Parameter			
**Class of interest**	**Precision**	**True positive rate**	**Specificity**	**F1**
Background	1.00	0.97	1.00	0.98
Intratubular casts	0.84	0.87	0.99	0.85
Tubular necrosis	0.99	0.84	1.00	0.91
Regenerating epithelium	0.71	0.46	1.00	0.56
Adipose tissue	0.90	0.91	0.97	0.91
Glomeruli	0.99	0.91	1.00	0.95
Proximal tubules	0.99	0.85	1.00	0.92
Distal tubules/Collecting ducts	0.77	0.91	0.96	0.83
Transitional epithelium	0.94	0.94	1.00	0.94
Stroma	0.72	0.96	0.95	0.83

Biology Open • Supplementary information
